# Change of mammographic density predicts the risk of contralateral breast cancer - a case-control study

**DOI:** 10.1186/bcr3451

**Published:** 2013-07-22

**Authors:** Maria EC Sandberg, Jingmei Li, Per Hall, Mikael Hartman, Isabel dos-Santos-Silva, Keith Humphreys, Kamila Czene

**Affiliations:** 1 Department of Medical Epidemiology and Biostatistics, Karolinska Institutet, Nobels väg 12B, Stockholm, 177 71, Sweden; 2 Human Genetics, Genome Institute of Singapore, 60 Biopolis St, Singapore, 138672, Singapore; 3 Saw Swee Hock School of Public Health, National University of Singapore, 16 Medical Drive Singapore, 117597, Singapore; 4 Department of Surgery, National University of Singapore, 1E Kent Ridge Road, Singapore, 119228, Singapore; 5 Department of Non-Communicable Disease Epidemiology, London School of Hygiene and Tropical Medicine, London, Keppel Street, London WC1E 7HT, United Kingdom

**Keywords:** Contralateral breast cancer, mammographic density, risk, breast density, epidemiology

## Abstract

**Introduction:**

Mammographic density is a strong risk factor for breast cancer, but it is unknown whether density at first breast cancer diagnosis and changes during follow-up influences risk of non-simultaneous contralateral breast cancer (CBC).

**Methods:**

We collected mammograms for CBC-patients (cases, N = 211) and unilateral breast cancer patients (controls, N = 211), individually matched on age and calendar period of first breast cancer diagnosis, type of adjuvant therapy and length of follow-up (mean follow-up time: 8.25 years). The odds of CBC as a function of changes of density during follow-up were investigated using conditional logistic regression, adjusting for non-dense area at diagnosis.

**Results:**

Patients who experienced ≥10% absolute decrease in percent density had a 55% decreased odds of CBC (OR = 0.45 95% CI: 0.24 to 0.84) relative to patients who had little or no change in density from baseline to first follow-up mammogram (mean = 1.6 (SD = 0.6) years after diagnosis), whereas among those who experienced an absolute increase in percent density we could not detect any effect on the odds of CBC (OR = 0.83 95% CI: 0.24 to 2.87).

**Conclusion:**

Decrease of mammographic density within the first two years after first diagnosis is associated with a significantly reduced risk of CBC, this potential new risk predictor can thus contribute to decision-making in follow-up strategies and treatment.

## Introduction

Mammographic density is one of the strongest risk factors for breast cancer; a meta-analysis of 14,000 cases and 226,000 non-cases showed that the women with >75% mammographic density have almost five times the risk of breast cancer compared to women in the lowest density group (<5%) [1]. Mammographic density has also been shown to be important for breast cancer recurrence [2] and survival [3]. Several hormonal factors affect mammographic density and changes of density have been shown to be associated with pharmacological therapies, such as hormone replacement therapy [4] and tamoxifen [5].

Despite the well-known and strong association between mammographic density and unilateral breast cancer, the effect of mammographic density on the risk of a second primary breast cancer in the opposite breast, contralateral breast cancer (CBC), has to our knowledge not been investigated before. Breast cancer patients have approximately double the risk of CBC, compared to healthy women's risk of breast cancer [6] and this increased risk does not seem to decline with time after first diagnosis [7-9]. This translates into 10 to 15% of all breast cancer patients being diagnosed with CBC within 20 years of initial diagnosis [10,11]. When investigating hormonal risk factors for unilateral breast cancer no association with risk of CBC has been identified [12-14]. Trends of breast cancer incidence and breast cancer mortality indicate that CBC will be a greater clinical challenge in the future, since the population at risk of CBC is increasing [15]. Since CBC also has a far less well characterized risk profile [8] and considerably worse prognosis than unilateral breast cancer, new tools for prediction of CBC would be of great clinical importance [16].

The question of whether decreasing mammographic density is associated with a decreased future risk of breast cancer, or not, has been investigated in several observational studies. Two showed an association [17,18] and one did not [19]. The question was also examined in a randomized trial of tamoxifen among healthy women at high risk of breast cancer, for which the estimated association was more pronounced among the tamoxifen treated, although the association (albeit non-statistically significant) was also seen among the women who received placebo [20]. If this association were also present among breast cancer patients, for the risk of CBC, this would have important clinical implications for follow-up care. The aim of this matched nested case-control study was, therefore, to assess whether change of mammographic density after the first breast cancer diagnosis predicts a change in risk of CBC.

## Methods

### Study population

The study was nested within the catchment population of the Stockholm Breast Cancer Register, a population-based register of all breast cancer patients diagnosed since 1976 in the Stockholm-Gotland health-care region (N >30,000). Women with invasive CBC diagnosed more than one year after the first invasive cancer and with an available mammogram close to the first diagnosis (N = 458) were identified as potential cases. Patients with invasive unilateral breast cancer in the same register were identified as potential controls. Women with a first primary cancer other than breast cancer and women with distant metastasis at the first or second breast cancer diagnosis were excluded in order to minimize the risk of the CBC being a misclassified metastasis. Further, second primary breast cancers can obviously also occur in the same breast as the first cancer; ipsilateral breast cancer. We chose not to include these cancers in the present study since also these cancers are less likely to be primary cancers. For each case, one control was randomly selected and matched to the corresponding case on the calendar period of the first breast cancer diagnosis (+/- two years), age at the first breast cancer diagnosis (+/- two years), adjuvant therapy and follow-up time, so that the control had survived without distant metastasis or CBC at least as long as the time between the first and second cancer for the corresponding case, a strategy known as density sampling [21]. From the Stockholm Breast Cancer Register we retrieved information on menopausal status at the time of the (first) breast cancer diagnosis, estrogen receptor (ER) status and tumor-node-metastasis (TNM)-stage of the (first) cancer, in addition to the matching variables. From the medical records of the cases and controls we retrieved information on hormone replacement therapy at the time of the (first) breast cancer diagnosis as well as additional information on menopausal status and ER-status.

We collected baseline and follow-up mammograms for the cases and controls. The baseline mammogram was defined as a mammogram from the contralateral breast, that is, the breast not affected by cancer, taken at any time during the year prior to diagnosis, or within two weeks after diagnosis, of the first cancer. Follow-up mammograms were defined as mammograms from the unaffected, contralateral breast taken at least one year, but no more than five years, after diagnosis of the first cancer. We used the first available mammogram in the defined time period. We defined sets of two individuals comprising one CBC case and one matched control (unilateral breast cancer patient) and a total of four mammograms (one baseline and one follow-up mammogram for each patient) of the same view. The media-lateral-oblique (MLO) view of mammograms have been the preferred view in the Swedish screening program [22] and were, therefore, used for our primary selection. Sets of cranial-caudal (CC)-mammograms made up 14% of the final sample.

For 99 of the 458 eligible CBC-cases we could not locate any follow-up mammogram and for 88 of the CBC-cases either the baseline or the follow-up mammogram could not be used (for example, due to low quality of the mammogram), while for 271 patients (59%) both the baseline and at least one follow-up mammogram of the unaffected breast from the same view was assessable and could be used. Among these patients we could trace the baseline and follow-up mammograms from the correct side and view for the corresponding control in 211 cases. These 211 case-control sets were thus included in the analysis sample. The CBC patients excluded due to lack of eligible mammograms did not differ from those included in the analysis in relation to age or calendar period of first diagnosis.

For comparison with the risk of CBC as a function of baseline mammographic density (that is, density at the time of first breast cancer diagnosis), we also examined the risk of unilateral breast cancer in relation to mammographic density. To achieve this, for each unilateral breast cancer case, we also measured the mammographic density of a healthy woman. The healthy controls (N = 142) were randomly selected from a breast cancer case-control study, extensively described elsewhere [23], for which all available mammograms had been previously collected. The mammograms of the healthy controls were matched to the unilateral breast cancer patients by calendar period and age of the corresponding unilateral breast cancer patient at her first diagnosis.

The mammograms were digitized using an Array 2905HD Laser Film Digitizer (Array Corporation, Tokyo, Japan), which covers a range of 0 to 4.7 optical density. The density resolution was set at 12-bit spatial resolution. Mammographic density was measured using our automated thresholding method [24], which incorporates the knowledge of a trained observer by using measurements obtained by an established user-assisted threshold method - Cumulus [25] - as training data. The externally validated results showed a high correspondence between our automated method and the established user-assisted thresholding method Cumulus (r_percent mammographic density_) = 0.88 (95% CI: 0.87 to 0.89).

### Statistical analysis

We estimated the percent mammographic density as well as the absolute size of the dense area and of the total area of the breast. Percent density and dense area have been used in previous studies and have both been shown to be important predictors of breast cancer risk [26]. For descriptive purposes we calculated the mean changes of mammographic density (unadjusted) in different groups of study participants.

As the first step, the risk of CBC as a function of baseline mammographic density was analyzed using conditional logistic regression, contrasting CBC patients to unilateral breast cancer patients. Further, by the same type of analysis we then contrasted the unilateral breast cancer patients to healthy controls. Percentage density at baseline was categorized into ≤5%, >5 to 25% (reference level), >25 to 50% and >50%; these cutoffs have been used extensively [1]. Dense area at baseline was categorized into: ≤20 cm^2^, >20 to 40 cm^2 ^(reference level), >40 to 60 cm^2 ^and >60 cm^2^, with these categories corresponding approximately to quartiles of the baseline dense area distribution. Calculating also the total breast area enabled us to adjust for non-dense area in the analyses; adjustment for this variable has recently been shown to be preferential to adjusting for body mass index (BMI) [27]. All analyses were thus adjusted for age and calendar period of diagnosis, adjuvant therapy and follow-up time (through matching) and also for non-dense area at first diagnosis, categorized in quartiles. Trend tests were carried out based on ordered categories of percent density/dense area.

For our main analysis, conditional logistic regression was used for analyzing risk of CBC as a function of "change" of mammographic density from baseline to first follow-up mammogram, categorized in three levels: absolute decrease ≥10%, stable (-10% to +10%, reference level) and absolute increase ≥10%, in agreement with previous literature [20]. Further, we investigated change of density in terms of absolute dense area, also categorized in three levels: ≥10 cm^2 ^reduction, stable (-10 cm^2 ^to +10 cm^2^, reference level) and ≥10 cm^2 ^increase in dense area. Both analyses were adjusted through matching for age and calendar period of first diagnosis, first adjuvant therapy and follow-up time. In an additional model, we made further adjustments for baseline non-dense area and baseline mammographic density (percent density when using change in percent density and dense area when using change in dense area), both categorized in quartiles. Patients with <10% or >90% percent mammographic density at baseline (N = 66), or those with <10 cm^2 ^or >70 cm^2 ^dense area (N = 84), were excluded, since they cannot possibly undergo changes in percent mammographic density, or dense area, of the defined magnitude. A similar strategy has been used by others when studying changes in mammographic density [5]. Trend test was performed using the ordinal categories of change of percent density and area density, respectively.

Finally, as an exploratory analysis we stratified our population on menopause status at first breast cancer to investigate the effect of change of mammographic density on the risk of CBC in the two subgroups, adjusting for non-dense area at baseline and percent density at baseline. Case-control pairs discordant for menopause status (N = 35) was not included in this analysis.

All data preparation and analyses were carried out using SAS Statistical Package 9.2 (SAS Institute Inc., Cary, NC, USA). This study was approved by the Ethical Review Board at Karolinska Institutet, Stockholm, Sweden. As no contact was made with the study persons and the data were analyzed anonymously, informed consent was not obtained. This exception from informed consent was confirmed by the ethical committee.

## Results

A total of 422 subjects (211 cases and 211 controls) were included in the analysis (Table 1). The mean time from diagnosis to follow-up mammogram was 1.6 years, 90% of the follow-up mammograms were taken between 1 and 2.2 years after diagnosis of the first breast cancer and there was no difference between cases and controls. The mean breast density at baseline was 28%.

**Table 1:** Distribution of CBC case and unilateral breast cancer controls

		Cases	Controls	*P*-value*
**Age at (first) diagnosis (%)**	≤45 years	37 (18)	37 (18)	
	45 to 55 years	68 (32)	68 (32)	
	55 to 65 years	56 (27)	56 (27)	
	≥65 years	50 (24)	50 (24)	-
	
**Calendar period of (first) diagnosis (%)**	1976 to 1980	31 (15)	30 (14)	
	1981 to 1985	40 (19)	41 (19)	
	1986 to 1990	45 (21)	41 (19)	
	1991 to 1995	51 (24)	49 (23)	
	1996 to 2005	44 (21)	50 (24)	-
	
Adjuvant therapy (%)*	No adjuvant therapy	39 (18)	39 (18)	
	Radiotherapy only	57 (27)	57 (27)	
	Endocrine therapy	87 (41)	87 (41)	
	Chemotherapy	28 (13)	28 (13)	-
	
**Mean follow-up time in years**		8.25	8.25	-
**Percent density at (first) diagnosis (%)**	≤5%	13 (6)	11 (5)	
	5 to 25%	87 (41)	87 (41)	
	25 to 50%	97 (46)	87 (41)	
	≥50%	14 (7)	26 (12)	0.23
	
	Quartiles:			
**Dense area at (first) diagnosis (%)**	≤20 cm^2^	55 (26)	55 (26)	
	20 to 34 cm^2^	44 (21)	56 (27)	
	34 to 53 cm^2^	56 (27)	49 (23)	
	≥53 cm^2^	56 (27)	51 (24)	0.79
	
**Change in percent density until first **	Absolute decrease (>10%)	40 (19)	56 (27)	
**follow-up mammogram (%)**	Stable density	164 (78)	143 (68)	
	Absolute increase (>10%)	7 (3)	12 (6)	0.07
	
**Change in dense area until first **	Absolute decrease (>10 cm^2^)	61 (29)	75 (36)	
**follow-up mammogram**	Stable density	131 (62)	113 (54)	
	Absolute increase (>10 cm^2^)	19 (9)	23 (11)	0.21
	
	Quartiles:			
**Non-dense area at (first) diagnosis (%)**	≤67 cm^2^)	42 (20)	60 (28)	
	67 to 93 cm^2^	58 (27)	43 (20)	
	93 to 127 cm^2^	50 (24)	55 (26)	
	≥127 cm^2^	61 (29)	53 (25)	0.10
	
**Mean time until first follow-up mammogram in years (SD)**		1.56 (0.59)	1.54 (0.57)	0.56
	
Menopause status at diagnosis (%)**	Premenopausal	89 (42)	84 (40)	
	Postmenopausal	119 (56)	124 (59)	0.62

Subdivided by matching variables (age at diagnosis, calendar period of diagnosis, adjuvant therapy and follow-up time), exposure variables (dense area at diagnosis and change of dense area), potential confounding variables (non-dense area at diagnosis and time to first follow-up mammogram), and stratifying variable (menopause status).

* P-value for Chi-square test of association when testing categorical variables and for Student's t-tests when testing the continuous variable (mean time until first follow-up mammogram).

*Endocrine therapy may be with or without radiotherapy, chemotherapy may be with or without radiotherapy and/or endocrine therapy.

** Six patients had uncertain menopause status (for example, hysterectomy).

Table 2 describes the mean change of mammographic density, measured in absolute percent density, from baseline to follow-up mammogram. The change is similar over the calendar period and over different categories of total breast area. As expected, the mammographic density decreases significantly more in the women diagnosed before menopause, compared to women diagnosed after menopause (*P*-value <0.01).

**Table 2:** Mean absolute change of percent density (PD) from baseline until first follow-up mammogram

	N	Meandecrease (%-units)	95% CI for mean decrease of PD	*P*-value
**Total**	422	-3.94	-4.89, -3.00	-
**CBC case/control status**				0.09
**Cases**	211	-3.13	-4.39, -1.87	
**Controls**	211	-4.75	-6.16, -3.34	
**Age at time of (first) cancer:**				<0.01
**<45 years**	74	-5.30	-8.39, -2.22	
**45 to 54 years**	136	-5.85	-7.61, -4.10	
**55 to 64 years**	112	-4.13	-5.60, -2.65	
**≥65 years**	100	**-0.13**	**-1.50, 1.25**	
**Calendar period of (first) cancer:**				0.25
**1976 to 1980**	62	-4.11	-7.57, -0.66	
**1981 to 1985**	80	-3.78	-6.24, -0.32	
**1986 to 1990**	90	-2.27	-4.09, -0.46	
**1991 to 1995**	102	-3.91	-5.70, -2.12	
**1996 to 2005**	88	-5.71	-7.26, -4.16	
**Total breast area at baseline:**				0.27
**Smallest quartile**	103	-2.54	-4.66, -0.41	
**2^nd ^quartile**	105	-4.68	-6.71, -2.66	
**3^rd ^quartile**	110	-4.91	-6.75, -3.07	
**Largest quartile**	104	-3.56	-5.16, -1.96	
**Adjuvant therapy of (first) cancer:**				<0.01
**No adjuvant therapy**	78	-1.09	-3.09, 0.92	
**Radiotherapy only**	114	-3.22	-5.14, -1.30	
**Endocrine therapy (with/without radiotherapy)**	174	-4.15	-5.37, -2.93	
**Chemotherapy (with/without other adjuvant therapy)**	56	-8.74	-5.24, -12.24	
Menopause status at (first) cancer:*				<0.01
**Premenopausal**	173	**-5.90**	**-7.70, -4.10**	
**Postmenopausal**	243	**-2.56**	**-3.54, -1.58**	
Postmenopausal HRT use:*				<0.01
**with Current use of HRT at diagnosis**	51	**-6.55**	**-8.90, -4.19**	
**with No current use of HRT at diagnosis**	127	**-1.66**	**-2.90, -0.43**	
**ER-status of (first) cancer:**				0.63
**ER-positive**	295	-4.15	-5.29, -3.01	
**ER-negative**	53	-3.46	-5.92, -0.99	
TNM-stage of (first) cancer:*				0.67
**1**	244	-4.06	-5.27, -2.85	
**2**	157	-3.48	-5.10, -1.86	
**3**	16	-5.36	-11.19, 0.47	

**The mean change is calculated for cases and control combined**.

* Six (1%) women with unknown menopause; 65 (27%) postmenopausal women with unknown HRT. Five women with unknown TNM-stage.

TNM, tumor-node-metastasis

We found no association between mammographic density at baseline and risk of CBC using either percent mammographic density or dense area (*P*-value for trend: 0.40 and 0.96 for percent density and dense area, respectively) (Table 3). Also, when mammographic density was analyzed as a continuous measure no effect was found; odds ratio (OR) for percent density: 1.00 (95% CI: 0.98 to 1.01) and for dense area: 1.00 (95% CI: 0.99 to 1.01). We further compared the baseline density between unilateral breast cancer patients and healthy controls. As expected, we found a statistically significant increasing risk of breast cancer with increasing mammographic density (*P*-values for trend: <0.01 and 0.02 for percent density and dense area, respectively).

**Table 3:** Odds ratio of CBC and unilateral breast cancer in relation to levels of mammographic density

Density at diagnosis	**CBC-patients vs**. unilateral breast cancer patients			Unilateral breast cancer patients vs. healthy women		
**Percent density**	**N**	**OR**	**95% CI**	**N**	**OR**	**95% CI**
**≤5% **	24	1.28	0.52 to 3.13	19	0.58	0.20 to 1.69
**>5 to 25%**	174	1.00	Ref.	125	1.00	Ref.
**>25 to 50%**	184	1.14	0.71 to 1.82	111	2.27	1.18 to 4.37
**>50%**	40	0.46	0.17 to 1.21	32	2.89	1.01 to 8.21
*P*-value for trend*		0.40			<0.01	
**Area density**	**N**	**OR**	**95% CI**	**N**	**OR**	**95% CI**
**≤20 cm^2^**	106	1.35	0.79 to 2.32	85	0.65	0.36 to 1.17
**>20 to 40 cm^2^**	144	1.00	Ref.	101	1.00	Ref.
**>40 to 60 cm^2 ^**	96	1.25	0.71 to 2.19	52	1.40	0.69 to 2.83
**>60 cm^2 ^**	76	1.27	0.70 to 2.29	49	1.58	0.76 to 3.26
*P*-value for trend*		0.96			0.02	

* Trend test is based on ordered categories of percent density and dense area, respectively.

Analysis of the risk of contralateral breast cancer (CBC) is adjusted by CBC-patients being matched to unilateral breast cancer patients by age at diagnosis, calendar period of diagnosis, follow-up time and adjuvant therapy. The analysis of risk of unilateral breast cancer is adjusted by unilateral breast cancer patients being matched to healthy women by age at mammogram and calendar period of mammogram In addition to matching all models are also adjusted for non-dense area at baseline mammogram. The mammographic density was measured at the time of the first breast cancer for CBC-cases and unilateral controls, mammographic density of the healthy controls were measured in the same calendar period and at the same age as the corresponding control with unilateral breast cancer.

When analyzing change of mammographic density, we observed a 55% lower risk of CBC for women with an absolute decrease in mammographic density of ≥10% from baseline to follow-up mammogram, compared to women with stable mammographic density (OR = 0.45 (95% CI: 0.24 to 0.84)) (Table 4). We found no statistically significant effect of increasing absolute mammographic density compared to stable density (OR = 0.83 (95% CI: 0.24 to 2.87)). Through the matched case-control design, these findings are independent of age and calendar period of first diagnosis, adjuvant therapy and follow-up time. The adjustments for non-dense area at baseline and percent density at baseline affected the estimates only marginally, but these variables are included since they are potential confounders. Using absolute dense area as a measure of mammographic density we found a similar effect of 46% risk decrease for women with ≥10 cm^2 ^decrease from baseline to follow-up mammogram, compared to women with stable density (OR = 0.54; 95% CI: 0.30 to 0.99) (Table 4). No statistically significant effect was seen for ≥10 cm^2 ^increase of density compared to women with stable density (OR = 0.71 (95% CI: 0.30 to 1.69)). Further, adjustment for hormone replacement therapy affected the estimates only marginally (OR for ≥10% decrease = 0.41 (95% CI: 0.21 to 0.78), OR for ≥10% increase = 0.87 (95% CI: 0.25 to 3.07)) and is not included in the models. Finally, we stratified our analysis on treatment groups; unfortunately, only two groups had large enough power, the patient group treated with radiotherapy only and the patient group treated with endocrine therapy (with or without radiotherapy). The association between decreasing mammographic density (measured as percent density) and risk of CBC was similar in the two groups; OR = 0.52 (95% CI: 0.18 to 1.51) for the endocrine therapy group and OR = 0.43 (95% CI: 0.14 to 1.28) for the radiotherapy group (data not shown in Table).

**Table 4:** Odds ratio of CBC in relation to changes in mammographic density after first breast cancer

Post-diagnostic change of density					
Percent density	N	OR*	95% CI	OR**	95% CI
**Absolute decrease ≥10%**	96	**0.49 **	**0.28 to 0.85**	**0.45 **	**0.24 to 0.84**
**Stable (<10% decrease to <10% increase)**	243	1.00	Ref.	1.00	Ref.
**Absolute increase ≥10%**	17	0.74	0.23 to 2.40	0.83	0.24 to 2.87
*P*-value for trend***		0.04		0.04	
**Area density**	**N**	OR*	**95% CI**	OR**	**95% CI**

**Absolute decrease ≥10 cm^2^**	108	0.67	0.38 to 1.16	**0.54 **	**0.30 to 0.99**
**Stable (<10 cm^2 ^decrease to <10 cm^2 ^increase)**	197	1.00	Ref.	1.00	Ref.
**Absolute increase ≥10 cm^2^**	33	0.79	0.35 to 1.78	0.71	0.30 to 1.69
*P*-value for trend***		0.35		0.13	

* Adjusted through matching for age at diagnosis, calendar period of diagnosis, adjuvant therapy and follow-up time.

** Adjusted through matching for age at diagnosis, calendar period of diagnosis, adjuvant therapy and follow-up time, and additionally in the analyses for percent density and non-dense area at first diagnosis.

*** Trend test is based on the ordered categories of change of percent density and dense area, respectively

Patients with <10% or >90% percent mammographic density at baseline (N = 66), or those with <10 cm^2 ^or >70 cm^2 ^dense area (N = 84), were excluded, since they cannot possibly undergo changes in percent mammographic density, or dense area, of the defined magnitude A similar strategy has previously been used by others when studying changes in mammographic density [5].

As an exploratory analysis of the effect of change of mammographic density on CBC, we stratified our population on menopause status at baseline mammogram. Among the premenopausal women an absolute decrease of mammographic density of 10% or more from baseline to follow-up mammogram was associated with an OR of CBC of 0.29 (95% CI: 0.09 to 0.92 (N = 164)) compared to the reference level of stable density. The corresponding OR for post-menopausal women was 0.49 (95% CI: 0.16 to 1.45) (N = 188). The effect of density reduction and menopausal status was not statistically significant (*P*_interaction _= 0.47).

Finally, we stratified the mammograms by view; CC-view (19%) and MLO-view (81%). We found very similar estimates for the effect of decreasing mammographic density on the risk of CBC in the two groups, although the statistical significance was lost in the CC-view group (OR for MLO-group: 0.45 (95% CI: 0.22 to 0.91), OR for the CC-group: 0.42 (95% CI: 0.06 to 2.77)).

## Discussion

To our knowledge, this is the first study to have investigated the risk of CBC as an effect of mammographic density at the time of diagnosis of the first cancer and of its subsequent changes. In a population-based setting we estimated that an absolute decrease of mammographic density from diagnosis to first follow-up mammogram of at least 10% confers a significant 55% decrease in risk of CBC. In contrast, mammographic density at diagnosis does not seem to predict risk of CBC.

Strengths of the study include its ability to investigate both baseline density and changes after first diagnosis and the use of both absolute (dense area) and relative (percent density) measures of mammographic density. The study is limited mainly by its small size, resulting from the large proportion of cases that had to be excluded from the analysis because the required mammograms could not be traced. Exclusion of patients due to unavailability of mammograms is a potential source of bias if the missingness is differential; it, however, seems unlikely that the mammograms should be missing on the basis of mammographic density. Further, unavoidably, access to the patient's mammograms is a requirement for studies of mammographic density. Reassuringly, the excluded patients did not differ from those included in the analysis in relation to important factors such as age at first diagnosis (*P*-value: 0.23) and calendar period of first diagnosis (*P*-value: 0.12). There were, however, differences with respect to adjuvant therapy for the first cancer; the included patients had received radiotherapy and endocrine therapy to a somewhat larger degree (radiotherapy; 29% vs. 23% for excluded patients, endocrine therapy; 39% vs. 29% for excluded patients). Since the study was matched for treatment, this difference has potential implications only for the generalizability of our results, as the patients receiving no adjuvant therapy and the patients receiving chemotherapy are somewhat under-represented in this study. In general, the availability of the mammograms in Sweden during the study period is primarily driven by archiving policies, rather than patients not having mammograms taken.

In contrast to the effect of mammographic density on the risk of unilateral breast cancer among healthy women, mammographic density at baseline did not seem to influence the risk of CBC in breast cancer patients. None of the categories of mammographic density conferred any statistically significant change in risk from the reference group. The fact that some of the OR estimates were decreased is most likely due to low power in those specific categories (*P*-value for trend: 0.40) (Table 3). The lack of association for mammographic density at baseline is a somewhat unexpected finding but mimics the effect of hormonal/reproductive factors, which increases the risk of breast cancer [28,29] but not the risk of CBC [12-14]. An alternative explanation for the lack of association between mammographic density and risk of CBC could be that there was a systematic difference in mammographic density of the unilateral breast cancer patients selected as controls for the current study, compared to unilateral breast cancer patients in general. To investigate this concern we studied the effect of mammographic density on the risk of breast cancer in unilateral breast cancer patients compared to healthy women and reassuringly found the expected strong association between mammographic density and the risk of breast cancer.

Our study showed that women who experienced at least 10% decrease in mammographic density after the first breast cancer diagnosis were at a substantially lower risk of developing CBC than those women whose mammographic density remained stable (Table 4). Two previous studies have investigated the relation between change of mammographic density and risk of unilateral breast cancer and showed that decreasing density was associated with a decreasing risk of developing breast cancer [17,18]. However, no previous study has examined changes in density after a first diagnosis of breast cancer in relation to risk of CBC. Not only are CBC patients a selected subgroup of women with high susceptibility to breast cancer, they are on average younger and have a higher prevalence of family history of the disease. Furthermore, a large proportion of CBC patients are treated with adjuvant therapy for their first breast cancer. In a primary prevention study, women treated with tamoxifen or placebo showed a decreased risk of breast cancer following a decrease in mammographic density [20], the effect was stronger among the tamoxifen-treated patients, but present also among the non-treated, though not statistically significantly so, indicating the presence of also other mechanisms, not mediated through tamoxifen. In the present study, we found that the decrease in the risk of CBC associated with a decline in mammographic density was independent of the type of adjuvant treatment administered; when stratifying on adjuvant therapy we did not see any indication of different effects in different treatment groups, but these analyses are associated with low power.

Kerlikowske *et al. *[17] suggested that the effect of change in mammographic density on the risk of unilateral breast cancer might be more pronounced in premenopausal women. We stratified our analysis on menopausal status at first breast cancer diagnosis and although we had low numbers for this analysis we still found a suggestion of a stronger association between decreased density and risk of CBC in the premenopausal women. The majority of the premenopausal women diagnosed with breast cancer will go through menopause relatively soon, either naturally, due to aging, or artificially, due to adjuvant chemotherapy [30], and the change in mammographic density resulting from menopause is relatively large [31]. The findings in the pre-menopausal group, in combination with the findings from the analysis stratified on therapy, indicate that a decrease in mammographic density, regardless of the mechanism, might result in a subsequent decrease in CBC-risk.

## Conclusion

Our findings indicate that women who experience ≥10% absolute decrease in mammographic density from the first diagnosis until the first follow-up mammogram (approximately 1.6 years later) decrease their risk of CBC to about half. Furthermore, the association between decreasing mammographic density and risk of CBC was independent of therapy given for the first cancer. The 10% cutoff has been previously shown as the minimum change that could be reproducibly detected visually [20] and might, therefore, be clinically useful. In the present study, 23% of the participating women experienced such a decrease. If confirmed, change of mammographic density can be used to predict the risk of CBC, and can thus contribute to decision-making in follow-up routines and adjuvant treatment regimens.

## Abbreviations

BMI: body mass index: CBC: contralateral breast cancer; CC: cranial-caudal; CI: confidence interval; ER: estrogen receptor; MLO: media-lateral-oblique; OR: odds ratio; SD: standard deviation; TNM stage: tumor-node-metastasis stage

## Competing interests

The authors declare that they have no competing interests.

## Authors' contributions

MECS participated in the design of the study and in data collection, performed the statistical analysis, and drafted the manuscript. JL carried out the density measurements and revised the manuscript for important intellectual content. PH, MH, IdSS and KH conceived the study, participated in its design and revised the manuscript for important intellectual content. KC conceived, designed and coordinated the study and helped in drafting the manuscript. All authors read and approved the final manuscript.
